# Einthoven Dissertation Prizes 2022

**DOI:** 10.1007/s12471-022-01698-4

**Published:** 2022-05-13

**Authors:** J. J. Piek

**Affiliations:** Department of Cardiology, Heart Centre, Amsterdam University Medical Centres, location Academic Medical Centre, University of Amsterdam, Amsterdam, The Netherlands

The Einthoven Dissertation Prize is named after Willem Einthoven, a pioneer in cardiovascular medicine. He recorded the first human electrocardiogram in 1902, for which he was awarded the Nobel Prize in 1924. The annual Einthoven Dissertation Prize is an initiative of the Netherlands Heart Institute (*NHI*) and the Netherlands Society of Cardiology (*NVVC*) to select the top three cardiovascular theses.

This year, the jury received a total of 23 PhD dissertations for selection. Their ranking was based upon a combination of parameters that included the curriculum vitae of the candidate, and the scientific originality of the PhD thesis and its relevance for the cardiovascular field. Moreover, several objective bibliometric parameters were used, which included the number of articles in citation index journals, both in PubMed and the Web of Science (WoS), the number of citations in WoS, the Hirsch index and finally the contributions of the candidate as first author.

Based upon this evaluation, the jury selected the following nominees: Lisette van der Does (Erasmus Medical Centre Rotterdam), Diederik van der Feen (University Medical Centre Groningen), and Job Verdonschot (Maastricht University Medical Centre+). The members of the jury were Prof. Pieter A.F.M. Doevendans (NHI), Dr Joan G. Meeder (NVVC), Dr Marco J.W. Götte (*CardioVasculair Onderwijs Instituut*), Dr Martin E.W. Hemels (*Werkgroep Cardiologische centra Nederland*) and Dr Gerrit Veen (President *Concilium Cardiologicum*). The three candidates presented their PhD theses at the Scientific Spring Meeting of the NVVC in the Netherlands, on 21 April 2022. We congratulate the laureates for their excellent scientific work and their presentations during the meeting.

## Summary: Mapping of atrial fibrillation: back to the drawing board

Despite years of research and much controversy among scientists, the mechanism underlying persistent atrial fibrillation (AF) is still mainly unknown, and as a result, ablative therapies frequently fail. Mapping techniques in clinical practice are not detailed enough to understand the exact mechanism underlying AF. We studied new techniques to map electrical conduction of the atria in great detail and discovered a new mechanism explaining persistence of AF.

A high-density electrode array with 192 electrodes was sequentially placed on the entire atria of patients undergoing cardiac surgery. This was proven to be a safe way to map atrial activation during both sinus rhythm and AF. Using this mapping approach, we observed that even during sinus rhythm, differences in conduction disturbance patterns were present between patients with different valvular heart diseases and between patients with and without AF. We then recorded, for the first time in humans, simultaneous electrical activity in high resolution from both sides of the right atrial wall and discovered that the endocardial and epicardial layers of the right atrial wall are asynchronously excited during AF.

Fourteen patients with AF demonstrated between 1% and 56% of endo-epicardial asynchrony of the right atrium, especially those with persistent AF for over a year (all > 20%). Endo-epicardial asynchrony creates opportunity for transmural conduction of AF waves and thereby the emergence of new AF waves on the other side that maintain AF. In our study, 65% of these new emerging waves could be attributed to asynchrony and transmural conduction of fibrillation waves. Excitable tissue in three dimensions exponentially increases opportunities for the maintenance of AF waves. Asynchrony also occurs more frequently during atrial extrasystolic beats than during normal sinus beats, and asynchrony up to 130 ms was observed in extra-atrial beats. Atrial extrasystolic beats have the potential to start atrial arrhythmia, and asynchrony could contribute to their arrhythmogenic potential.

One ablative therapy for persistent AF is ablation of complex fractionated atrial electrograms. However, eleven different definitions for electrogram fractionation were found in a literature search, and many pathophysiological causes of fractionation exist. We determined that the fractionated signals on the electrogram mainly originate from remote electrical activity, including asynchronous endo-epicardial activation.

Last, we studied the use of fractionation as a way to identify asynchronous activation of the atrial wall based on electrogram recordings from only one side of the wall, which makes the detection easier in clinical practice. The sensitivity of fractionation-based detection of asynchrony in patients during sinus rhythm was high (90%). Unipolar electrograms were more suitable for asynchrony detection than bipolar electrograms due to a larger signal-to-noise ratio of fractionated signals and less disturbance of additional fractionated signals.

Detailed mapping of conduction disorders will help us to better understand AF and to develop treatment strategies that are better tailored to each patient. Mapping techniques in clinical practice will require a high resolution and a mode to detect endo-epicardial asynchrony in order to discover and adequately treat the underlying mechanism of persistent AF.

*Lisette van der Does* ^1,2^

^1^ Department of Cardiology, Erasmus Medical Centre, Rotterdam, The Netherlands

^2^ Department of General Practice, Julius Centre for Health Sciences and Primary Care, University Medical Centre Utrecht, The Netherlands

lisettevanderdoes@gmail.com

## Summary: A window for reversibility in pulmonary arterial hypertension

Pulmonary arterial hypertension (PAH) is a lethal arteriopathy characterised by progressive pulmonary vascular rarefaction, occlusion, degeneration and sclerosis. Most forms of PAH are not diagnosed until the arteriopathy has reached an advanced disease stage, which is irreversible, meaning that neither PAH-targeted therapy nor treatment of the causally associated comorbidity can reverse the disease. In PAH associated with congenital heart disease (PAH-CHD), there is a unique reversible phase, in which haemodynamic unloading through *timely* shunt closure can completely reverse the disease. Unfortunately, this curative potential is lost beyond a certain ‘point of no return’, after which progressive vascular remodelling occurs despite shunt correction. This implies the existence of a biological mechanism that specifically accounts for the transition from a potentially reversible PAH phenotype towards a progressive, irreversible one. It is currently unknown why PAH in time is irreversible or how loss of reversibility can be recognised in this or any other PAH aetiology.

For this thesis, the clinical entities of reversible and irreversible PAH-CHD were explored, as well as the pathological effects of disturbed blood flow on the pulmonary circulation. Using a translational approach, we then applied these findings to model the biological response to haemodynamic unloading and the transition towards irreversible PAH in rats with a shunt. We hypothesised that the specific factors that drive irreversibility can be adopted as targets for new pharmacological interventions that may reverse ‘irreversible’ vascular remodelling in end-stage PAH. Such treatments are currently unavailable.

Using shunt-induced PAH in rats, we demonstrated a phase-dependent dichotomous reversibility response to haemodynamic unloading, similar to human PAH-CHD. This enabled us to identify and compare vascular profiles of reversible and irreversible PAH, based on RNA sequencing. We cumulatively reported that loss of reversibility is associated with a switch from a proliferative to a senescent vascular phenotype and that this process occurs in the context of genotoxic stress and a deranged DNA damage response. To support our concept that cellular senescence is not only associated with but also causal to the irreversible nature of end-stage PAH, we targeted senescence using the senolytic ABT-263. This reversed the haemodynamic and structural changes associated with severe PAH refractory to haemodynamic unloading in vivo. We also found evidence for pulmonary vascular regeneration after targeted senolysis in rats.

To provide a therapy more directly applicable in the clinic, we evaluated apabetalone (RVX-208) in a multicentre preclinical trial. Apabetalone is a BET bromodomain inhibitor that counteracts BRD4, an epigenetic ‘super-enhancer’ protein that modulates chromatin during persistent genotoxic stress and enables transcription of numerous proproliferative, prosurvival and proinflammatory genes involved in PAH. BRD4 also induces the inflammatory secretory phenotype of senescent cells. At a clinically relevant dose, apabetalone normalised the PAH phenotype in endothelial and smooth muscle cells and reversed vascular remodelling in two complementary PAH rat models. In an additional rat model for right ventricular failure, apabetalone was shown to be safe for the pressure-loaded right ventricle and enhanced its physiological adaptation. A clinical trial with apabetalone is currently ongoing.

*Diederik E. van der Feen*

Centre for Congenital Heart Diseases, University Medical Centre Groningen, The Netherlands (Current position: Department of Pulmonology, Spaarne Gasthuis, Haarlem, The Netherlands)

diederikvanderfeen@gmail.com

## Summary: Causes and consequences of dilated cardiomyopathy: integrating genotype and phenotype to redefine disease diagnostic and therapeutics

Dilated cardiomyopathy (DCM) is defined as the presence of left ventricular or biventricular dilatation and systolic dysfunction in the absence of abnormal loading conditions or coronary artery disease sufficient to cause global systolic dysfunction. The clinical definition of DCM is based solely on the presence of abnormal cardiac structure and function. DCM is treated as a monomorphic disease with standard heart failure therapy, with the aim to restore cardiac function. This approach does not capture the extensive aetiological heterogeneity of DCM. Pathogenic gene variants, acquired disease and a combination of both can all lead to DCM. For a significant subgroup of DCM patients, a genetic aetiology has been established. A pathogenic gene variant has been found in ~ 20% of DCM patients, and genetic analysis is considered a first-tier test in DCM. Most guidelines recommend genetic testing based on familial forms of DCM in the absence of acquired disease. However, studies performing genetic testing in unselected cohorts of DCM patients are scarce.

In this thesis, we explored the contribution of the genotype to the phenotype and integrated this information in order to identify more homogeneous clinical subgroups of DCM to improve treatment and prognosis. First, we collected extensive clinical data in a large cohort of genotyped DCM patients to detect clinical characteristics of patients with a pathogenic gene variant (Part I). Second, we used machine learning approaches to integrate the data of genetics with echocardiographic, cardiac magnetic resonance imaging, electrophysiological, laboratory and biopsy data to create a new classification of DCM patients while focusing on clinical utility. By analysing transcriptomics data, we aimed to identify the underlying mechanism that drives disease progression in these patients (Part II). Last, we focused on the implications of genetic testing of a patient’s family members and evaluated methods used to screen these relatives for cardiac disease (Part III).

Using a gene panel consisting of 48 genes, the overall genetic yield was 19% in an unselected, consecutive DCM cohort, irrespective of a family history of DCM or an acquired disease. Overall, the presence of a pathogenic genetic variant was negatively associated with treatment response, except for the titin (*TTN*) variants. Pathogenic variants are the most prevalent in *TTN* and are characterised by arrhythmias and increased interstitial fibrosis but also by a relatively mild disease course with a favourable treatment response. Patients with truncating variants in filamin C (*FLNC*) have a high risk of ventricular arrhythmias and cardiac sudden death. The arrhythmic risk of *TTN* and *FLNC* before left ventricular dysfunction develops, warrants collective efforts to evaluate multiparametric risk stratification models.

We detected a specific transcriptomic profile of *LMNA* and *TTN* cardiomyopathies and linked it to the clinical phenotype to define a more malignant subphenotype of *TTN*. Using unsupervised machine learning approaches, we identified four phenogroups within DCM: two of which represented opposite poles of severity, and the other two represented distinct clinical subgroups with a high event rate irrespective of cardiac function. Interestingly, we were able to create a decision tree only comprising four commonly used clinical variables to classify a DCM patient in one of these four subgroups, which had significant prognostic relevance. Also, the transcriptomic profiles of the patients in such a subgroup were homogeneous, indicating a comparable disease mechanism and a possible treatment target.

Finally, we showed that global longitudinal strain analysis of echocardiographic images has a high sensitivity to detect early cardiac abnormalities in relatives of DCM patients who have an otherwise normal left ventricular ejection fraction.

This thesis describes the clinical consequences of genetic testing in DCM (Part I), integrates the genetic results with other aetiologies and phenotypic manifestations to refine diagnosis and direct treatment of the disease (Part II) and describes the impact of genetics on (first-degree) relatives of a DCM patient (Part III).

*Job Verdonschot* ^1,2^

^1^ Department of Clinical Genetics, Maastricht University Medical Centre+, Maastricht, The Netherlands

^2^ Department of Cardiology, Cardiovascular Research Institute Maastricht, Maastricht, The Netherlands

job.verdonschot@mumc.nl

Job Verdonschot won first prize, Diederik van Feen second prize and Lisette van der Does third prize.

